# A Study of the effect of eigenvalues on the Moore Gibson Thompson model under photoacoustic excitation of semiconductors

**DOI:** 10.1038/s41598-025-03266-3

**Published:** 2025-05-27

**Authors:** A. El-Dali, Mohamed I. A. Othman, Esraa M. Gamal, Soliman Alkhatib

**Affiliations:** 1 Faculty of Science, Department of Mathematics, Helwan University, Cairo, 11795 Egypt; 2 Department of Mathematics, Faculty of Science, Zagazig University, Zagazig, 44519 Egypt; 3 College of Computer Information Technology, American University in the Emirates (AUE), Dubai, 503000 UAE

**Keywords:** Moore-Gibson-Thompson model, Photo-acoustic waves, Semiconductor, Photo-thermal excitation, Eigenvalue approach, Materials science, Mathematics and computing, Optics and photonics

## Abstract

Regarding this investigation, the Moore-Gibson-Thompson (MGT) model was developed with the impact of acoustic pressure. This research’s light is spotted on semiconductor material undergoing thermo-acoustic and optical deformation in the context of a theory of photo-thermoelasticity (PTE). The governing equations are formulated using a modified photo-excitation model, where (MGT) equation represents the heat conduction during processes of optical transport. This model represents the coupling between plasma, thermal, mechanical-elastic, and acoustic wave propagation. Analytical solutions for the main physical quantities are obtained utilizing the Laplace transform method combined with the vector–matrix differential equation method. Boundary conditions for the acoustic, plasma, and thermo-mechanical effects are applied at the outer surface of the medium. Numerical inversion of Laplace transforms is performed to obtain complete space–time solutions for primary fields. Silicon is utilized as a representative semiconductor material for numerical computations, with the results presented graphically and discussed with various influencing parameters. This study is significant because it provides a novel way to analyze the behavior of semiconducting materials under photo-acoustic excitation, applying the eigenvalue approach to a system previously modeled using simple methods. It fills existing gaps in the literature related to the application of the MGT model in semiconducting photo-acoustics and provides more detailed and reliable predictions for real-world applications.

## Introduction

The traditional theory of thermoelasticity has several generalizations. One relaxation period for the thermoelastic process is introduced in the first generalization, known as the Lord-Shulman (L-S) theory, which was put up by Lord and Shulman^1^. Green and Lindsay (G-L)^2^ created the second generalization, considering two relaxation time parameters. Different models classified as type I, type II, and type III are presented in the third generalization introduced by Green and Naghdi^3^. In its linearized form, the G-N I model is equivalent to the traditional coupled thermo-elasticity theory; energy dissipation is not permitted in the G-N II model, but it isallowed in the G-N III model, where the heat flux combinestype I and type II.

In recent years, the (MGT) theory in thermoelasticity originated as a refinement of classical thermoelastic and wave propagation models. Initially developed to address limitations in earlier theories, such as Fourier’s law of heat conduction, the MGT theory offers a more accurate description of heat conduction and wave phenomena. One of the key issues with classical models, like the one proposed by Fourier, is an assumption of infinite speed for thermal signal propagation, which contradicts physical reality. The MGT theory introduces heat propagation of a finite speed by incorporating higher-order time derivatives and thermal relaxation terms. This theory is instrumental in describing heat and wave interactions in thermoelastic media, where thermal and mechanical effects are coupled. It finds applications in areas that require precise modeling of wave behavior, such as materials science and engineering, where the classical models’ assumptions of infinite propagation speed are inadequate. By introducing these modifications, the MGT theory overcomes some paradoxes inherent in classical thermoelasticity. One may find more research on the modified couple stress theory (MCST) in^4–12^.

The eigenvalue method streamlines the analysis by transforming a system of differential equations into algebraic ones, facilitating the examination of the system’s qualitative behavior, such as its resonance frequencies and the impact of thermal stresses on the material’s mechanical properties. This approach is widely used in both static and dynamic problems of thermoelasticity. Kalkal et al.^13^ applied the eigenvalue approach to analyze a thick plate’s fractal-order two-phase viscoelastic thermoelastic problem. In the context of the three-phase-lag (TPL) model, Othman et al.^14^ used the eigenvalue approach to study the impact of rotation on a micropolar thermoelastic medium. The effects of starting load on a visco-thermoelastic rotating media reinforced with fibers were investigated by Othman et al.^15^. Using higher-order memory-dependent derivatives, Abouelregal et al.^16^ used the eigenvalue approach to study the thermoelastic response of a semi-infinite media exposed to a moving heat source. Numerous authors have used the subjective eigenvalue approach to solve numerous issues^17–26^.

Photo-Acoustic Semiconducting in thermoelasticity involves examining the interaction between light (photo), sound (acoustic), and thermal (thermoelastic) effects in semi-conducting materials. In this process, when a semiconductor is exposed to modulated light, it absorbs the energy, leading to localized heating and subsequent thermal expansion. This expansion generates acoustic waves, which can be detected and analyzed. Understanding these ideas is essential for several applications, such as thermal imaging, laser-based material processing, and non-destructive testing. Wavelength, amplitude, and frequency dictate the properties of acoustic waves, which are produced by mechanical vibrations in materials or air and are essential in physics, engineering, and medicine. Investigating acoustic waves provides valuable information about a material’s microstructure and mechanical characteristics^27^. The study of photo-acoustic effects in semiconducting materials in the setting of thermoelasticity has been advanced by several researchers^28–35^.

This research delves into the intricate relationship between the mathematical model (MGT) and photo-acoustic effects in semiconductors, presenting a comprehensive framework that bridges theoretical analysis and practical applications. The study introduces the eigenvalue approach and offers a novel perspective on semiconductor materials’ dynamic stability, oscillatory behavior, and resonance phenomena under external excitations. This approach enhances the understanding of material responses and establishes a robust mathematical foundation for exploring complex interactions that have yet to be fully addressed in previous studies.Dell’Oro and Pata^36^ and Marin et al.^37^ have all looked at a variety of (MGT) model thermoelasticity theory-related issues.

The novelty of this work lies in its ability to extend and refine existing models, providing a more accurate representation of photo-acoustic effects in semiconductors. Unlike traditional approaches, this study integrates advanced mathematical techniques to uncover subtle dynamic behaviors critical for developing next-generation semiconductor technologies. Furthermore, the insights gained from this research have significant implications for optimizing semiconductor-based devices, including improving the resolution and efficiency of photo-acoustic imaging systems, enhancing the performance of electronic components, and paving the way for innovative applications in renewable energy and advanced communication systems.

## Generalized formulations of the problem

In this study, we investigate the photo-acoustic behavior of semiconducting materials within the framework of (MGT) theory under the assumptions of linearity, homogeneity, and isotropic properties. We focus on the interplay of multiple wave types, thermal, plasma, and elastic, induced by photo-acoustic excitation. We consider a semiconductor medium subjected to an external photo-acoustic source, which generates a complex interaction between temperature variations (thermal wave)$\theta \,({\varvec{r}},t),$ carrier densityfluctuations (plasma wave)$N({\varvec{r}},t)$ acoustic pressure $P({\varvec{r}},t),$ and the acoustic displacement vector (elastic wave)$u({\varvec{r}},t).$ The medium is assumed to be free of any force. We utilize the constitutive stress–strain equations to describe the medium’s behavior under these conditions, which connect the state variables (stress, strain, temperature change, acoustic waves, and carrier density) with their respective fluxes. These equations are critical for deriving the differential equations governing the wave interactions in the semiconductor. The specific forms of the constitutive equations employed are derived from the general principles of thermo-elasticity and are detailed in references^38–40^.

1 $$\sigma_{ij} = \mu \left( {u_{i,j} + u_{j,i} } \right) + \left( {\lambda \;u_{i,i} - \gamma_{\theta } \,\theta \, - \gamma_{n} N} \right)\delta_{ij} ,\,\,\,\,\,\,\,\,\quad e_{ij} = \frac{1}{2}\left( {u_{i,j} + u_{j,i} } \right).$$

The following is an expression of the equation of motion in tensor form^9^:

2 $$\rho u_{i,tt} = \mu u_{i,jj} + \left( {\mu + \lambda } \right)e_{ij,j} - \gamma_{\theta } \theta_{,i} - \delta_{n} N_{,i} .$$

In the general situation, the relationship between thermal waves and plasma-acoustic dispersion can be expressed as^9^:

3 $$\frac{\partial N}{{\partial t}} = D_{E} N_{,ii} - \,\frac{N}{\tau } + \kappa \;\theta .$$

The interaction of plasma in a semiconductor medium produces acoustic waves, which in turn leads to the formulation of the coupled thermoacoustic equation, which is expressed as^41^:

4 $$P_{,ii} - \;\,\frac{1}{{C_{s}^{2} }}P_{,tt} - C_{r} \beta \theta_{,tt} = 0.$$

The heat conduction of classical Fourier’s law is fundamental for understanding heat. Transfer, as it reflects a temperature difference between two objects. Assuming constantthermal conductivity, Fourier’s law states that the amount of heat flux is directly proportional to the temperature differential. When there are no delays in heat propagation and the heat transfer rate is constant across time, a steady-state situation is where this linear equation stays true^42^.

5 $$q_{i} = - \;k\,\theta_{,j} .$$

The equation of heat conduction with plasma^43^:

6 $$\rho C_{e} \,\theta_{,t} + \;\gamma \,\theta_{0} e_{,t} - \;\frac{{E_{g} }}{\tau }N = - \;q_{i,j} .$$

According to Maxwell and Cattaneo’s theory, the equation of Fourier heat conduction is modified by adding relaxation time $\tau_{0}$, or thermal memory, as follows^44^:

7 $$\left( {1 + \tau_{0} \frac{\partial }{\partial t}} \right)q = - \,k\nabla \theta .$$

Green-Naghdi (GN-III)^45,46^ modifies the equation of heat conduction to include thermaldisplacement and rate of thermal conductivity, taking the following form^44^:

8 $$q = - \;\,\left[ {k\nabla \theta + k^{ * } \nabla \upsilon } \right].$$

Based on the relaxation time (thermal memory), where $\upsilon_{,t} = \theta ,$ the equation of heat conduction can be modified as^44^:

9 $$\left( {1 + \tau_{0} \frac{\partial }{\partial t}} \right)q = \left[ {k\nabla \theta + k^{*} \nabla \upsilon } \right].$$

The following can be used to obtain the equation of generalized heat conduction withphotothermal excitation based on Eqs. (7) through (9) and the MGT equation:

10 $$\left( {1 + \tau_{0} \frac{\partial }{\partial t}} \right)\left( {\rho C_{e} \frac{{\partial^{2} \theta }}{{\partial t^{2} }} + \gamma_{\theta } \theta_{0} \frac{{\partial^{2} e}}{{\partial t^{2} }} - \frac{{E_{g} }}{\tau }\frac{\partial N}{{\partial t}}} \right) = \left[ {k\nabla^{2} \frac{\partial \theta }{{\partial t}} + k^{*} \nabla^{2} \theta } \right].$$

The (L-S) and (GN-III) theories are generalized into the (MGPTE) theory^47^. The entire set of Eqs. (1) through (4) can be expressed as follows by taking the acoustic displacement vector in the direction during a one-dimensional photo-acoustic excitation:

11 $$\rho \,u_{,tt} = \left( {\lambda + 2\mu } \right)u_{,xx} - \delta_{n} N_{,x} - \gamma_{\theta } \theta_{,x} ,$$

12 $$N_{,t} = D_{E} N_{,xx} - \,\frac{N}{\tau } + \kappa \;\theta ,$$

13 $$P_{,xx} - \,\frac{1}{{C_{s}^{2} }}P_{,tt} - C_{r} \beta \theta_{,tt} \; = 0,$$

14 $$\left( {1 + \tau_{0} \frac{\partial }{\partial t}} \right)\left[ {\rho C_{e} \theta_{,tt} + \gamma_{\theta } \theta_{0} e_{,tt} - \frac{{E_{g} }}{\tau }N_{,t} } \right] = \left[ {k\frac{{\partial^{2} }}{{\partial x^{2} }}\theta_{,t} + k^{*} \theta_{,xx} } \right],$$

15 $$\sigma_{xx} = \sigma = (\lambda + 2\mu )u_{,x} - \delta_{n} N - \gamma_{\theta } \theta .$$

The following form of the non-dimensional can be investigated for more simplification for the system:

16 $$\left( {x^{\prime},\;u^{\prime}} \right) = \frac{{\left( {x,u} \right)}}{{C_{T} t^{*} }},\,\,\,\,\left( {t^{\prime},\tau^{\prime}_{0} } \right) = \frac{{\left( {t,\tau_{0} } \right)}}{{t^{*} }},\,\,\,\,\left( {\theta^{\prime},N^{\prime}} \right) = \frac{{\left( {\gamma_{\theta } \theta ,\delta_{n} N} \right)}}{2\mu + \lambda },\,\,\,\,\sigma ^{\prime} = \frac{\sigma }{\mu },\,\,\,\,e^{\prime} = e,\,\,\,P^{\prime} = \frac{P}{{P_{0} }}.$$

Rewriting the central system of equations using the dimensionless Eq. (16) and eliminating the prime notation results in:

17 $$\left( {\frac{{\partial^{2} }}{{\partial x^{2} }} - \,\frac{{\partial^{2} }}{{\partial t^{2} }}} \right)\,u - \frac{\partial \theta }{{\partial x}}\, - \,\frac{\partial N}{{\partial x}} = 0,$$

18 $$\left( {\frac{{\partial^{2} }}{{\partial x^{2} }} - A_{1} \frac{\partial }{\partial t} - A_{2} } \right)N + \varepsilon_{3} \,\theta = 0,$$

19 $$\left( {\frac{{\partial^{2} }}{{\partial x^{2} }} - \alpha_{p} \frac{{\partial^{2} }}{{\partial t^{2} }}} \right)P + \gamma_{p} \frac{{\partial^{2} \theta }}{{\partial t^{2} }}\, = 0,$$

20 $$\left( {k\frac{\partial }{\partial t} + k^{ * } t^{ * } } \right)\frac{{\partial^{2} \theta }}{{\partial x^{2} }} - \left( {1 + \tau_{0} \frac{\partial }{\partial t}} \right)\left( {\varepsilon_{1} \frac{{\partial^{2} \theta }}{{\partial t^{2} }} - \alpha_{2} \frac{\partial N}{{\partial t}} + \alpha_{3} \frac{{\partial^{2} e}}{{\partial t^{2} }}} \right) = 0,$$

21 $$\sigma \, = \, \sigma_{xx} = \alpha_{4} \,\left[ {\frac{\partial u}{{\partial x}} - (\theta \, + N)} \right],$$

where $$\,A_{1} = \frac{k}{{D_{E} \,\rho \,C_{e} }},\,\,\,\,A_{2} = \frac{{k\,t^{*} }}{{D_{E} \,\rho \,\tau \,C_{e} }},\,\,\,\,\,\alpha_{p} = \frac{{C_{T}^{2} }}{{C_{s}^{2} }},\,\,\,\,\gamma_{p} = \frac{{C_{r} \,\beta \,k\,C_{T}^{2} }}{{t^{*} \,C_{e} \,\gamma_{\theta } \,P_{0} }},\,\,\,\,\,\alpha_{2} = \frac{{\alpha_{T} \,E_{g} \,t^{*} \,k}}{{d_{n} \,\rho \,\tau \,C_{e} }},$$

$$\alpha_{3} = \frac{{\gamma_{\theta }^{2} \,\theta_{0} \,\,t^{*} }}{\rho },\,\,\,\,\alpha_{4} = \frac{2\mu + \lambda }{\mu },\quad \varepsilon_{1} = k,\,\,\,\,\varepsilon_{3} = \frac{{d_{n} \,k\,\kappa \,t^{*} }}{{\alpha_{T} \,\rho \,C_{e} \,D_{E} }}\,.\,\,\,\,$$

The parameters $\alpha_{3} ,$ $\alpha_{2}$ and $\,\varepsilon_{3}$ are the coupling between thermal and elastic effect, the coupling of thermal and energy effect, and the coupling between the thermal and electrical impact.

## Solving the problem

By applying the following formula^42^, the Laplace transformation approach effectively solves our problem by transforming partial differential equations (PDEs) to ordinary differential equations (ODEs) for any function:

22 $$L\left( {\aleph \left( {x,\,t} \right)} \right) = \overline{\aleph }\left( {x,\,s} \right) = \int\limits_{0}^{\infty } {\aleph \left( {x,\,t} \right)e^{ - st} \,d\,t} .$$

Under the subsequent starting circumstances and the system’s homogeneity.

$$\left. {u\left( {x,t} \right)} \right|_{t = 0} = \left. {\frac{{\partial u\left( {x,\,t} \right)}}{\partial t}} \right|_{t = 0} = 0,\left. {\theta \left( {x,t} \right)} \right|_{t = 0} = \left. {\frac{{\partial \theta \left( {x,\,t} \right)}}{\partial t}} \right|_{t = 0} = 0,$$

23 $$\left. {N\left( {x,t} \right)} \right|_{t = 0} = \left. {\frac{{\partial N\left( {x,\,t} \right)}}{\partial t}} \right|_{t = 0} = 0\,,\,\,\left. {\quad P\left( {x,t} \right)} \right|_{t = 0} = \left. {\frac{{\partial P\left( {x,\,t} \right)}}{\partial t}} \right|_{t = 0} = 0.$$

Assume the following boundary conditions to be accurate as well at $x = 0$

24 $$\sigma_{xx} \left( {0,t} \right) = b,\;b < 0,\quad N\left( {0,t} \right) = \frac{\raisebox{3pt}{-}\kern-5pt{\lambda}}{{D_{e} }},\quad \theta \left( {0,t} \right) = \theta_{0} ,\quad P\left( {0,t} \right) = P_{0} .$$

Using Eq. (20) with the starting conditions for the first four Eqs. (16) - (19), we derive:

25 $${\text{D}}^{2} \overline{u} = s^{2} \overline{u} + {\text{D}}\,\overline{\theta } + \,{\text{D}}\overline{N} ,$$

26 $$\,{\text{D}}^{2} \overline{N} = \alpha_{1} \overline{N} - \varepsilon_{3} \,\overline{\theta } ,$$

27 $${\text{D}}^{2} \overline{\theta } = \varepsilon_{1} \varepsilon_{2} s^{2} \overline{\theta } - \alpha_{2} \varepsilon_{2} s\,\overline{N} + \varepsilon_{2} \alpha_{3} s^{2} {\text{D}}\overline{u} \,,$$

28 $$\,{\text{D}}^{2} \overline{P} = \alpha_{p} s^{2} \overline{P} - \gamma_{p} s^{2} \,\overline{\theta } = 0,$$

29 $$\overline{\sigma }_{xx} = \alpha_{4} \left[ {{\text{D}}\overline{u} - \left( {\overline{\theta } + \overline{N} } \right)} \right],$$

where $${\text{D}} = \frac{d}{dx}\,,\,\,\,\,\,\,\,\,\varepsilon_{2} = \frac{{1 + \tau_{0} s}}{{k\,s + k^{ * } t^{ * } }},\,\,\,\,\,\,\,\,\alpha \,_{1} = A_{1} s\, + A_{2} ,\,\,\,\,\,\,\,\,\alpha_{4} = \frac{2\mu + \lambda }{\mu }.$$

The matrix form is utilized to get the solution of the system of Eqs. (12) – (14), by investigating the eigenvalues and eigenvectors to obtain the main physical variations as the following:

30 $$\frac{{{\text{d}}\vec{Z}}}{{{\text{d}}x}} = R\vec{Z},$$

where

31 $$\vec{Z} = \left[ {\begin{array}{*{20}c} {\begin{array}{*{20}c} {\begin{array}{*{20}c} u & N \\ \end{array} } & \theta & P & {\frac{du}{{dx}}} \\ \end{array} } & {\frac{dN}{{dx}}} & {\frac{d\theta }{{dx}}} & {\frac{dP}{{dx}}} \\ \end{array} } \right]^{T} ,\,$$

32 $$R = \left( {\begin{array}{*{20}c} 0 & 0 & 0 & 0 & 1 & 0 & 0 & 0 \\ 0 & 0 & 0 & 0 & 0 & 1 & 0 & 0 \\ 0 & 0 & 0 & 0 & 0 & 0 & 1 & 0 \\ 0 & 0 & 0 & 0 & 0 & 0 & 0 & 1 \\ {a_{51} } & 0 & 0 & 0 & 0 & {a_{56} } & {a_{57} } & 0 \\ 0 & {a_{62} } & {a_{63} } & 0 & 0 & 0 & 0 & 0 \\ 0 & {a_{72} } & {a_{73} } & 0 & {a_{75} } & 0 & 0 & 0 \\ 0 & 0 & {a_{83} } & {a_{84} } & 0 & 0 & 0 & 0 \\ \end{array} } \right),$$

where $$a_{51} = s^{2} ,\,\,\,\,\,\,\,a_{56} = a_{57} = 1,\,\,\,\,\,a_{62} = \alpha_{1} ,\,\,\,\,\,a_{63} = - \;\varepsilon_{3} ,\,\,\,\,\,\,\,a_{72} = - \;\varepsilon_{2} \alpha_{2} s,\,\,\,\,\,\,\,a_{73} = \varepsilon_{2} \varepsilon_{1} s^{2} ,$$

$$a_{75} = \;\varepsilon_{2} \alpha_{3} s^{2} ,\,\,\,\,\,\,\,\,a_{83} = - \;\gamma_{p} s^{2} ,\,\,\,\,\,\,\,\,\,a_{84} = \alpha_{p} s^{2} .$$

## A vector–matrix equation’s approach using eigenvalues and eigenvectors

Roots of the characteristic equation, which can be obtained from solving Eq. (30), represent the eigenvalues of the matrix $R$ can be set as $\lambda = \lambda_{1} ,$ $\lambda = \lambda_{2} ,$ $\lambda = \lambda_{3} ,$ $\lambda = \lambda_{4} ,$ $\lambda = \lambda_{5} ,$ $\lambda = \lambda_{6} ,$ $\lambda = \lambda_{7} ,$ $\lambda = \lambda_{8} ,$ where the characteristic equation can take the following form^9^:

33 $$\lambda^{8} - \Re_{1} \lambda^{6} + \Re_{2} \lambda^{4} - \Re_{3} \lambda^{2} + \Re_{4} = 0.$$

where

34 $$\left. \begin{gathered} \Re_{1} = ( - \;a_{51} - a_{62} - a_{73} - a_{57} a_{75} - a_{84} ) \hfill \\ \Re_{2} = \left( \begin{gathered} a_{51} a_{62} - a_{63} a_{72} + a_{51} a_{73} + a_{62} a_{73} + a_{57} a_{62} a_{75} - a_{56} a_{63} a_{75} + a_{51} a_{84} + a_{62} a_{84} \hfill \\ + a_{73} a_{84} + a_{57} a_{75} a_{84} \hfill \\ \end{gathered} \right) \hfill \\ \Re_{3} = \left( \begin{gathered} a_{51} a_{63} a_{72} - a_{51} a_{62} a_{73} - a_{51} a_{62} a_{84} + a_{63} a_{72} a_{84} - a_{51} a_{73} a_{84} - a_{62} a_{73} a_{84} \hfill \\ - \;a_{57} a_{62} a_{75} a_{84} + \;a_{56} a_{63} a_{75} a_{84} \hfill \\ \end{gathered} \right) \hfill \\ \Re_{4} = ( - \;a_{51} a_{63} a_{72} a_{84} + \;a_{51} a_{62} a_{73} a_{84} ) \hfill \\ \end{gathered} \right\}.$$

The corresponding eigenvectors can take the form $\vec{Q} = [q_{1} ,\,\,q_{2} ,\,\,q_{3} ,\,\,q_{4} ,\,\,q_{5} ,\,\,q_{6} ,\,\,q_{7} ,\,\,q_{8} ]^{\,T}$, according to the eigenvalues $\lambda_{\,i} (i = 1\,,\,\,2\,,\,\,3,\,\,4,\,\,5,\,\,6)$ which can be given as:

35 $$\left. \begin{gathered} q_{1} = - \lambda ( - \lambda^{2} + a_{73} )\left[ {a_{63} a_{56} - a_{57} ( - \lambda^{2} + a_{62} )} \right] \hfill \\ q_{2} = a_{63} (a_{51} - \lambda^{2} )(a_{73} - \lambda^{2} ) \hfill \\ q_{3} = a_{75} \lambda \left[ {a_{63} - a_{57} (a_{62} - \lambda^{2} )} \right] - a_{63} a_{72} (a_{51} - \lambda^{2} ) \hfill \\ q_{4} = \frac{{ - \;a_{83} }}{{(a_{84} - \lambda^{2} )}}\left[ {a_{75} \lambda \left[ {a_{63} a_{56} - a_{75} (a_{62} - \lambda^{2} )} \right]} \right] - a_{63} a_{72} (a_{51} - \lambda^{2} ) \hfill \\ q_{5} = \lambda \,q_{1} \hfill \\ q_{6} = \lambda \,q_{2} \hfill \\ q_{7} = \lambda \,q_{3} \hfill \\ q_{8} = \lambda \,q_{4} \hfill \\ \end{gathered} \right\}.$$

In this case, the vector solution of the physical variations of our problem can take the following form:

36 $$\overline{Z}(x,\,s) = \,\sum\limits_{i = 1}^{4} {B_{i} \overrightarrow {Q}_{i} \;e^{{ - \lambda_{i} x}} } .$$

Based on linearity, the primary physical fields are as follows:

37 $$\overline{u} = \sum\limits_{i = 1}^{4} {B_{i} \;Q_{i}^{1} e^{{ - \;\lambda_{i} x}} ,}$$

38 $$\overline{N} = \,\sum\limits_{i = 1}^{4} {B_{i} \,} Q_{i}^{2} e^{{ - \,\lambda_{i} x}} ,$$

39 $$\overline{\theta } = \,\sum\limits_{i = 1}^{4} {B_{i} \,} Q_{i}^{3} e^{{ - \,\lambda_{i} x}} ,$$

40 $$\overline{P} = \,\sum\limits_{i = 1}^{4} {B_{i} \,} Q_{i}^{4} e^{{ - \,\lambda_{i} x}} ,$$

41 $$\overline{\sigma } = \overline{\sigma }_{xx} = - \;\alpha_{4} \sum\limits_{i = 1}^{4} {B_{i} } (\lambda_{i} Q_{i}^{1} + Q_{i}^{2} + {\kern 1pt} Q_{i}^{3} )e^{{ - \lambda_{i} x}} .$$

## Boundary conditions

To determine the unknown functions $B_{i} ,\;(i = 1,2,3,4),$ we will implement the Laplace transform to the boundary conditions (24) as follows.

42 $$\overline{\theta }(0,s) = \frac{{\theta_{0} }}{s}\,\;\,,\,\,\,\overline{N}(0,s) = \,\;\frac{{N_{0} }}{s},\,\,\,\,\,,\,\,\,\,\,\overline{u}(0,s) = 0,\,\,\,\,\,\overline{\sigma }_{xx} (0,s) = \,\;0.$$

The method Cramer can determine the constants $B_{i}$ by using the following determinants^38^

43 $$\left. \begin{gathered} \Delta = \left| {\begin{array}{*{20}c} {Q_{1}^{5} } & {Q_{2}^{5} } & {Q_{3}^{5} } & {Q_{4}^{5} } \\ {Q_{1}^{2} } & {Q_{2}^{2} } & {Q_{3}^{2} } & {Q_{4}^{2} } \\ {Q_{1}^{3} } & {Q_{2}^{3} } & {Q_{3}^{3} } & {Q_{4}^{3} } \\ {Q_{1}^{4} } & {Q_{2}^{4} } & {Q_{3}^{4} } & {Q_{4}^{4} } \\ \end{array} } \right|,\,\,\,\quad \Delta_{1} = \left| {\begin{array}{*{20}c} 0 & {Q_{2}^{5} } & {Q_{3}^{5} } & {Q_{4}^{5} } \\ {N_{0} } & {Q_{2}^{2} } & {Q_{3}^{2} } & {Q_{4}^{2} } \\ {\theta_{0} } & {Q_{2}^{3} } & {Q_{3}^{3} } & {Q_{4}^{3} } \\ {P_{0} } & {Q_{2}^{4} } & {Q_{3}^{4} } & {Q_{4}^{4} } \\ \end{array} } \right|,\,\,\quad \Delta_{2} = \left| {\begin{array}{*{20}c} {Q_{1}^{5} } & 0 & {Q_{3}^{5} } & {Q_{4}^{5} } \\ {Q_{1}^{2} } & {N_{0} } & {Q_{3}^{2} } & {Q_{4}^{2} } \\ {Q_{1}^{3} } & {\theta_{0} } & {Q_{3}^{3} } & {Q_{4}^{3} } \\ {Q_{1}^{4} } & {P_{0} } & {Q_{3}^{4} } & {Q_{4}^{4} } \\ \end{array} } \right|,\, \hfill \\ \, \hfill \\ \Delta_{3} = \left| {\begin{array}{*{20}c} {Q_{1}^{5} } & {Q_{2}^{5} } & 0 & {Q_{4}^{5} } \\ {Q_{1}^{2} } & {Q_{2}^{2} } & {N_{0} } & {Q_{4}^{2} } \\ {Q_{1}^{3} } & {Q_{2}^{3} } & {\theta_{0} } & {Q_{4}^{3} } \\ {Q_{1}^{4} } & {Q_{2}^{4} } & {P_{0} } & {Q_{4}^{4} } \\ \end{array} } \right|,\quad \Delta_{4} = \left| {\begin{array}{*{20}c} {Q_{1}^{5} } & {Q_{2}^{5} } & {Q_{3}^{5} } & 0 \\ {Q_{1}^{2} } & {Q_{2}^{2} } & {Q_{3}^{2} } & {N_{0} } \\ {Q_{1}^{3} } & {Q_{2}^{3} } & {Q_{3}^{3} } & {\theta_{0} } \\ {Q_{1}^{4} } & {Q_{2}^{4} } & {Q_{3}^{4} } & {P_{0} } \\ \end{array} } \right|, \hfill \\ \end{gathered} \right\}$$

44 $$\left. \begin{gathered} Q_{i}^{1} = - \lambda_{i} (a_{73} - \lambda_{i}^{2} )[a_{56} a_{63} - a_{57} (a_{62} - \lambda_{i}^{2} )], \hfill \\ Q_{i}^{2} = a_{63} (a_{51} - \lambda_{i}^{2} )(a_{73} - \lambda_{i}^{2} ), \hfill \\ Q_{i}^{3} = a_{75} \lambda_{i} [a_{63} - a_{57} (a_{62} - \lambda_{i}^{2} )] - a_{63} a_{72} (a_{51} - \lambda_{i}^{2} ), \hfill \\ Q_{i}^{4} = \frac{{ - \;a_{83} }}{{(a_{84} - \lambda_{i}^{2} )}}[a_{75} \lambda_{i} [a_{63} a_{56} - a_{57} (a_{62} - \lambda_{i}^{2} )] - a_{63} a_{72} (a_{52} - \lambda_{i}^{2} )], \hfill \\ Q_{i}^{5} = - \;\lambda_{i} Q_{i}^{1} - Q_{i}^{2} - Q_{i}^{3} . \hfill \\ \end{gathered} \right\}.$$

In this case, the unknowns $B_{i} ,\;(i = 1,2,3,4),$ can be obtained as:

45 $$B_{1} = \frac{{\Delta_{1} }}{\Delta },\quad B_{2} = \frac{{\Delta_{2} }}{\Delta },\quad B_{3} = \frac{{\Delta_{3} }}{\Delta },\,\,\,B_{4} = \frac{{\Delta_{4} }}{\Delta }.$$

## Laplace transform inversion

One invaluable tool for determining the primary physical variations in the time domain is the inversion of the approach of Laplacetransformation.The sum of the Riemann approximation has been utilized to investigate the inversion of Laplace, greatly enhancing the accuracy of the results and letting you work out variations in the primary physical domains. Any function $\prod (x,\,t^{\prime})$ can be transformed from the frequency domain to the time domain, as described in^10,38^ provides more details about Laplace transform inversion.

46 $$\prod (x,\,t^{\prime}) = L^{ - 1} \{ \overline{\prod }(x,s)\} = \frac{1}{2\pi i}\int_{n - i\infty }^{n + i\infty } {e^{{st^{\prime}\,\,}} } \overline{\prod }(x,s)ds.$$

where $$n,\,m \in R,\,\,\,s = n + im,$$$$i = \sqrt {{\kern 1pt} - 1}$$, Eq. (44) can be rewritten as:

47 $$\prod (x,t^{\prime}) = \frac{{e^{nt} }}{2\pi }\,\int\limits_{ - \,\infty }^{\infty } {e^{i\beta t} \overline{\prod }(n + i\beta )d\beta .}$$

The expansion of Fourier is used to expand the variables for large integers, which are chosen free, in the interval $\left[ {0,\;2t^{\prime}} \right]$:

48 $$\prod (x,\,t) = \frac{{e^{{nt^{\prime}}} }}{{t^{\prime}}}\left[ {\frac{1}{2}\overline{\prod }\left( {x,\,n} \right) + {\text{Re}} \sum\limits_{k = 1}^{N} {\overline{\prod }\left( {x,\,n + \frac{ik\pi }{{t^{\prime}}}} \right)\left( { - 1} \right)^{n} } } \right],$$

where $Re$ is defined as the real part of the function.

## The discussion of the numerical results

The material chosen for the numerical simulation was silicon (Si). Table 1 contains silicon parameters^38^.

**Table 1:** Describe the parameter’s constants in the silicon (Si) SI units.

Symbol	Si	Unit
$\lambda$	$6.4 \times 10^{10}$	${\text{N/m}}^{{2}}$
$\mu$	$6.5 \times 10^{10}$	${\text{N/m}}^{{2}}$
$\rho$	$2330$	${\text{kg/m}}^{{3}}$
$T_{0}$	$800$	${\text{K}}$
$\tau$	$5 \times 10^{ - 5}$	${\text{s}}$
$D_{E}$	$2.5 \times 10^{ - 3}$	${\text{kg/m}}^{{3}}$
$E_{g}$	$1.11$	${\text{eV}}$
$d_{n}$	$- \;9 \times 10^{ - 31}$	${\text{m}}^{{3}}$
$\alpha_{t}$	$2.6 \times 10^{ - 6}$	${\text{K}}^{{ - {1}}}$
$C_{E}$	$695$	${\text{J/(kg}}\,{\text{.K)}}$
$k$	$150$	${\text{W}}\,.{\text{m}}^{{ - {1}}} {\text{.K}}^{{ - {1}}}$
$s$	$2$	${\text{m/s}}$
$C_{s}$	$8430$	${\text{m/s}}$
$\beta$	$2.56 \cdot 10^{ - 6}$	$^{^\circ } C$
$C_{r}$	$1.666$	
$\theta_{0}$	$1$	
$N_{0}$	$1$	

### The thermo-electric coupling parameter effect

This section presents numerical outcomes highlighting the influence of the thermo-electric coupling parameter $\varepsilon_{3}$ on various physical quantities (temperature, carrier density, displacement, acoustic pressure, stress, and strain). These results are obtained within the Moore-Gibson-Thompson (MGT) framework under photo-acoustic semi-conducting excitation conditions.

**Fig. 1:**
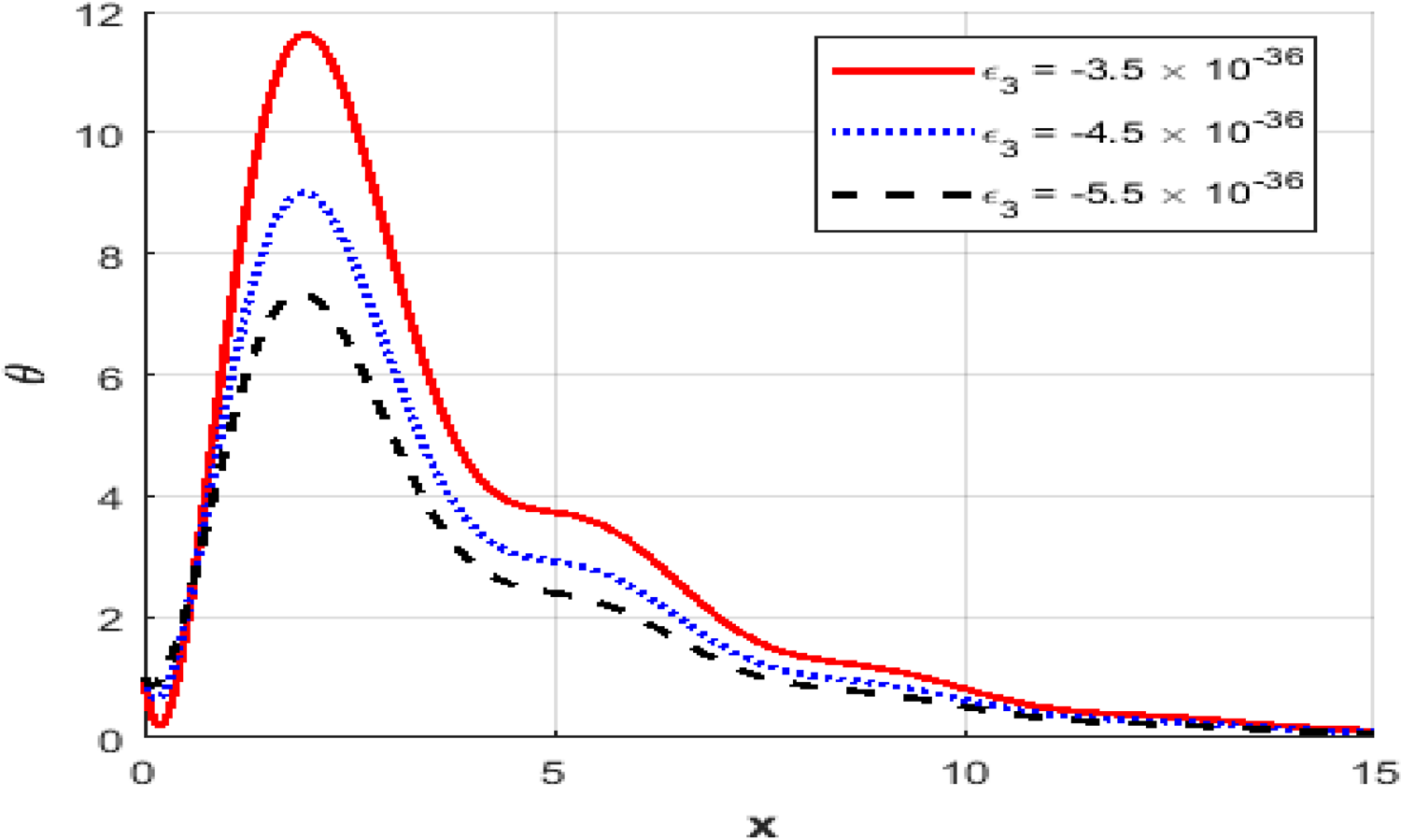
Variation of temperature $\theta$ against $x$ for different values of thermoelectric parameter.

**Fig. 2:**
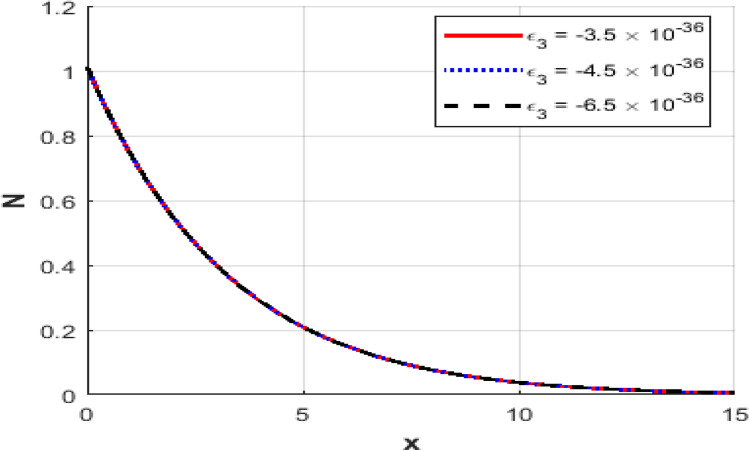
Variation of the carrier density $N$ against $x$ for different values of thermoelectric parameter.

**Fig. 3:**
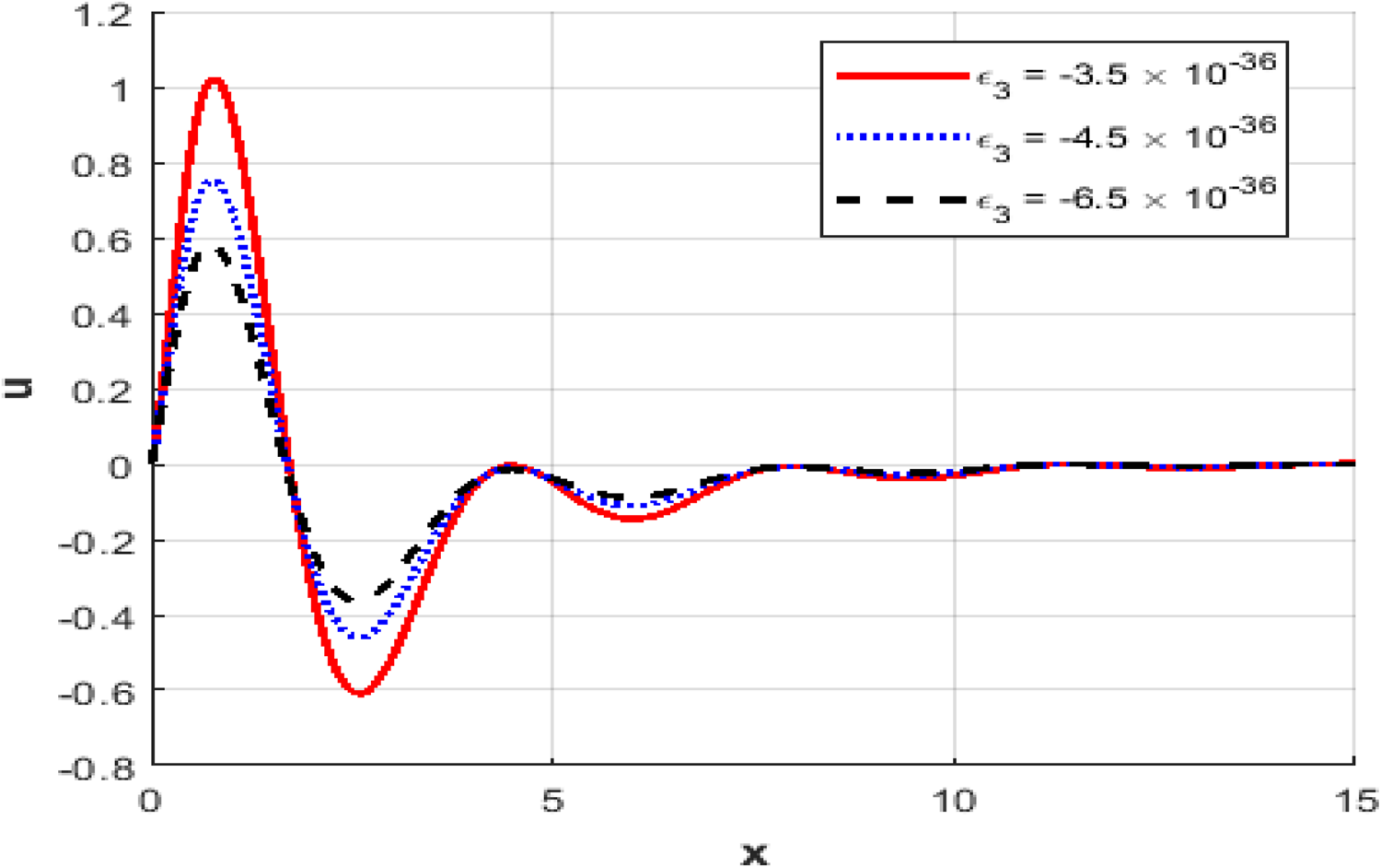
Variation of the displacement $u$ against $x$ for different values of thermoelectric parameter.

**Fig. 4:**
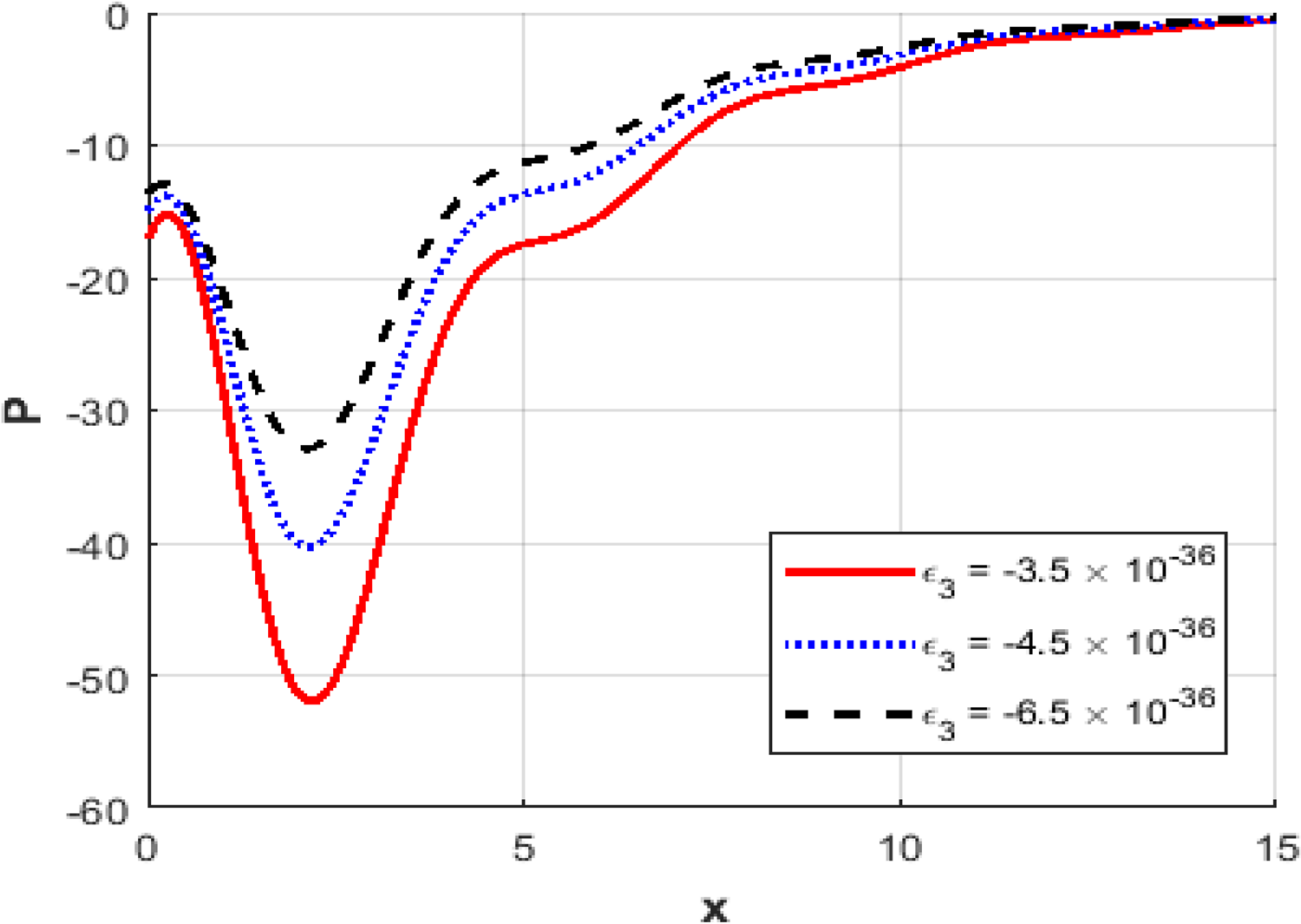
Variation of the acoustic pressure $P$ against $x$ for different values of thermoelectric parameter.

**Fig. 5:**
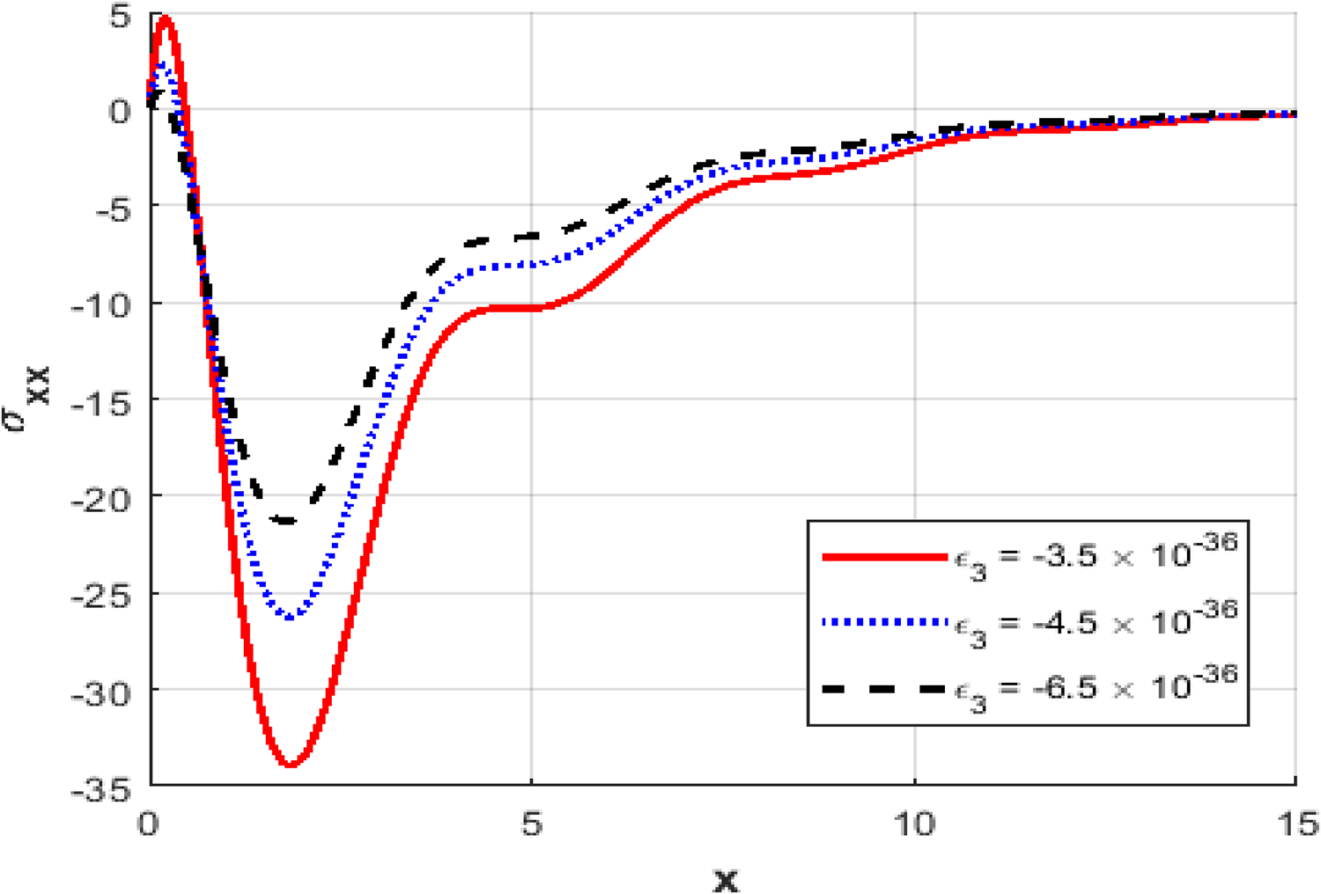
The stress distribution $\sigma_{xx}$ against $x$ for different values of thermoelectric parameter.

**Fig. 6:**
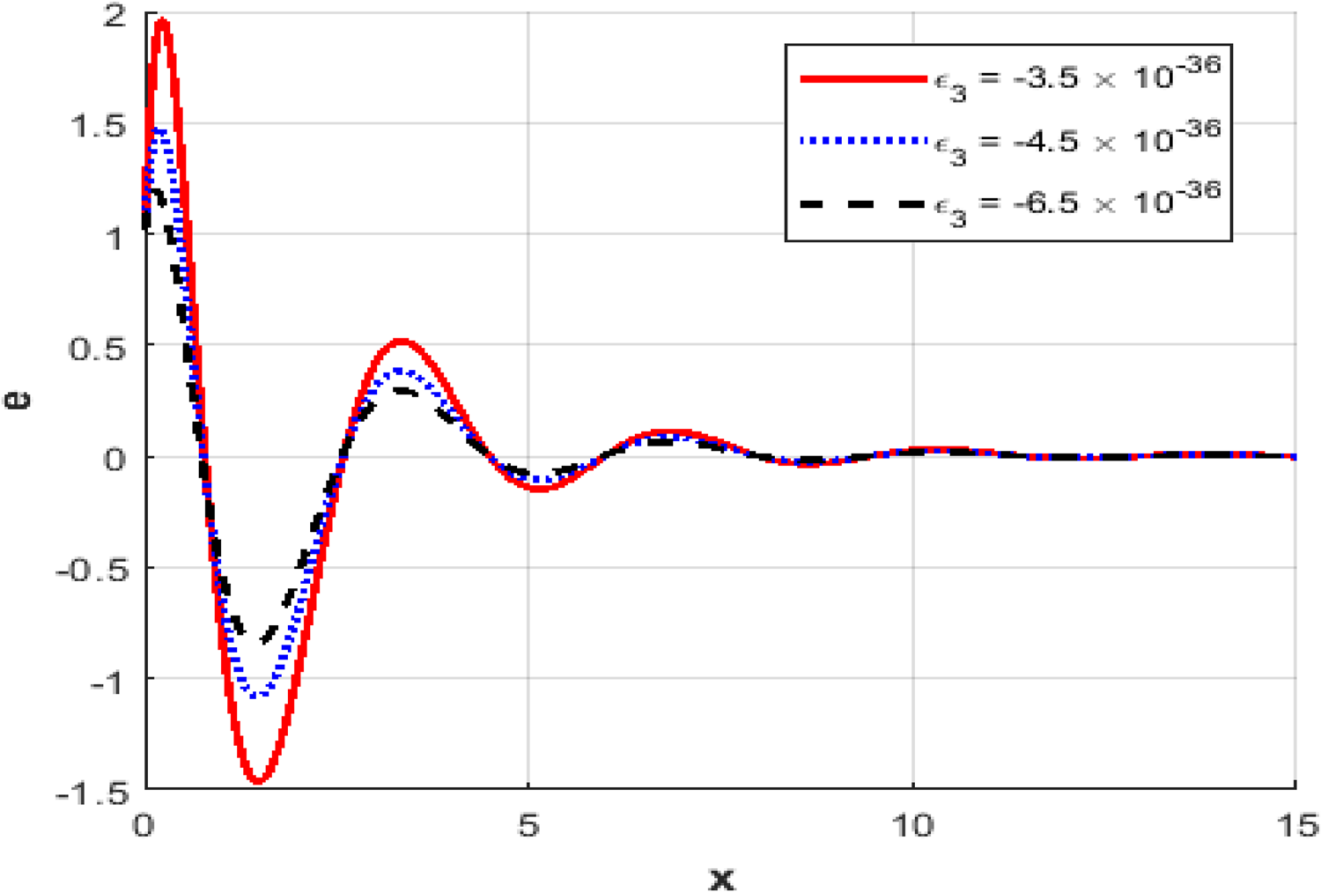
Variation of the strain $e$ against $x$ for different values of thermoelectric parameter.

Figure 1 represents the variation in temperature $T$ with position $x - axis$ for different values of the thermo-electric coupling parameter $\varepsilon_{3} .$ It is clear from the figure that an increase in the absolute magnitude of epsilon​three​ significantly reduces the peak temperature. For instance, the maximum temperature decreases noticeably as epsilon​three​ shifts from $- \;3.5*10^{ - \,36} {\text{to}} - 5.5*10^{ - \,36}$.Additionally, the temperature profile reveals a clear attenuation behavior as the spatial distance increases. In Fig. 2, the carrier density $N$ decreases monotonically with increasing distance $x - axis$, exhibiting a smooth exponential decay. Notably, varying the thermo-electric coupling parameter has a negligible influence on the carrier density profile, as evident from the overlapping curves. This indicates the insensitivity of carrier density distribution to changes in the coupling parameter under the given conditions. Figure 3 demonstrates the displacement $u$ as a function of position $x - axis$. The displacement shows a pronounced oscillatory behavior near the boundary, where amplitudes significantly depend on the thermo-electric coupling parameter. The magnitude of oscillations decreases as the absolute value of $\varepsilon_{3}$ increases, suggesting more excellent damping effects for larger coupling parameter magnitudes. At positions away from the boundary, displacement gradually attenuates towards zero. Figure 4 depicts the acoustic pressure $P$ profile. The acoustic pressure exhibits negative peaks near the boundary region, with magnitude strongly influenced by the thermo-electric coupling parameter. The most prominent peak in acoustic pressure occurs at $- \;3.5*10^{ - \,36}$, and decreasing the parameter’s magnitude reduces peak values and smooths the pressure distribution. The pressure eventually approaches zero as the distance from the source increases. The stress distribution $\sigma_{xx}$,as shown in Fig. 5, follows a trend like the acoustic pressure. Stress initially presents a significant negative peak whose magnitude diminishes substantially with an increased magnitude of the thermo-electric coupling parameter. The oscillatory pattern near the boundary region gradually attenuates towards zero, revealing stabilization in distant areas. Figure 6 shows the strain $e$ distribution, demonstrating prominent oscillatory patterns strongly modulated by changes in the thermo-electric coupling parameter. The amplitude of these oscillations significantly reduces as the magnitude of $\varepsilon_{3}$ becomes larger, reflecting enhanced damping. The strain progressively attenuates and stabilizes to negligible values further away from the excitation source.

### The thermo-elastic coupling parameter effect

**Fig. 7:**
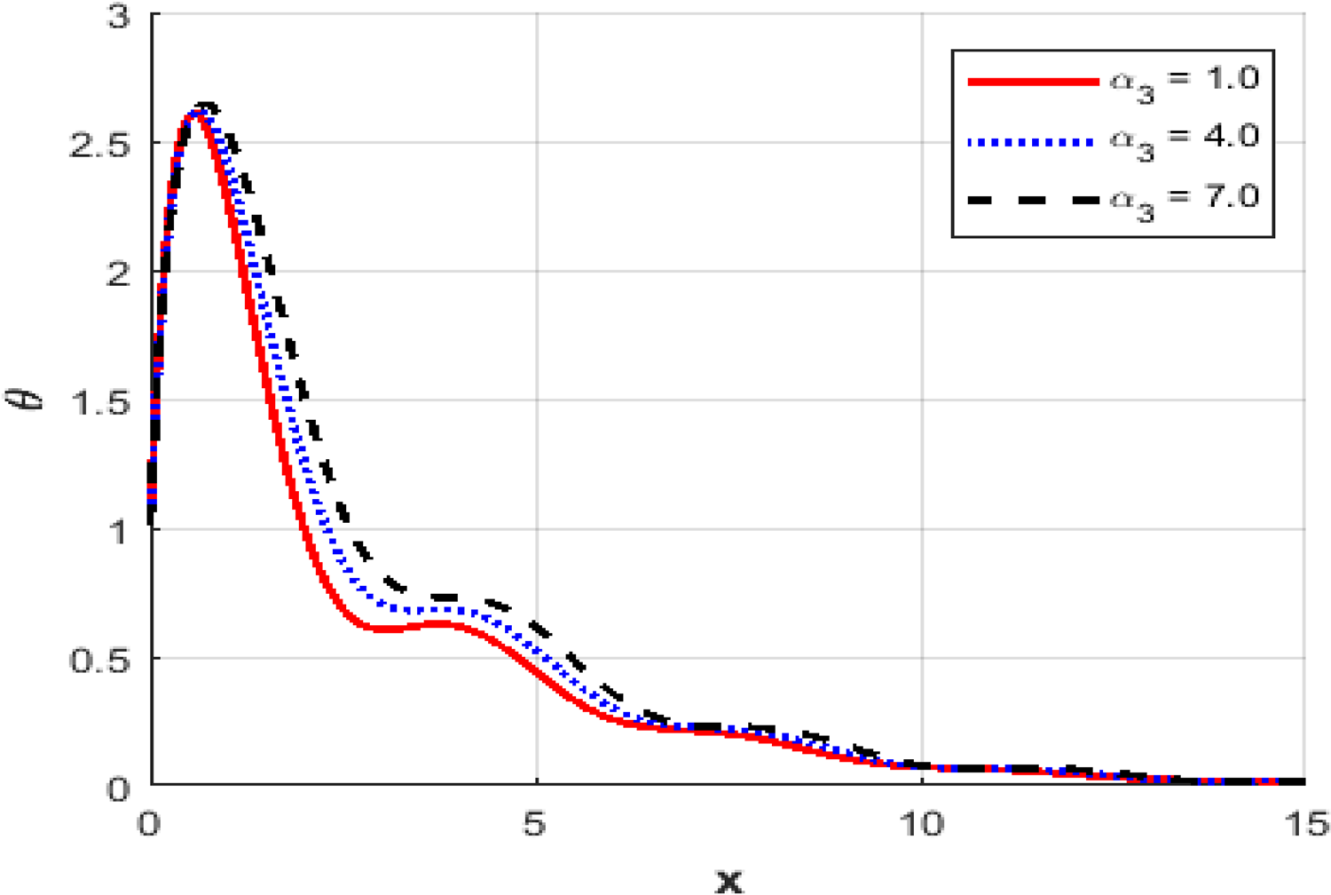
Variation of temperature $\theta$ against $x$ for different values of thermo-elastic parameter.

**Fig. 8:**
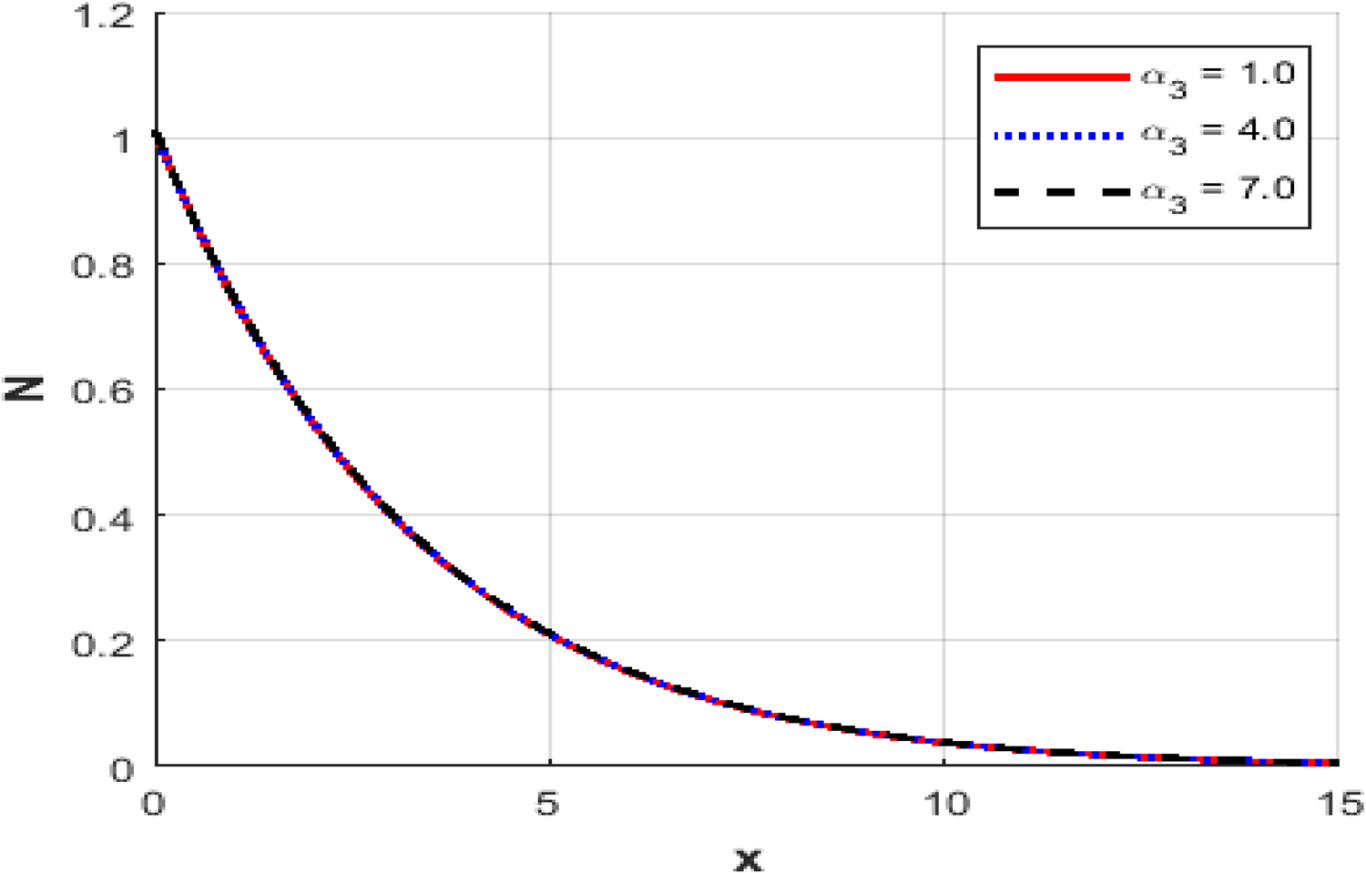
Variation of the carrier density $N$ against $x$ for different values of thermoelectric parameter.

**Fig. 9:**
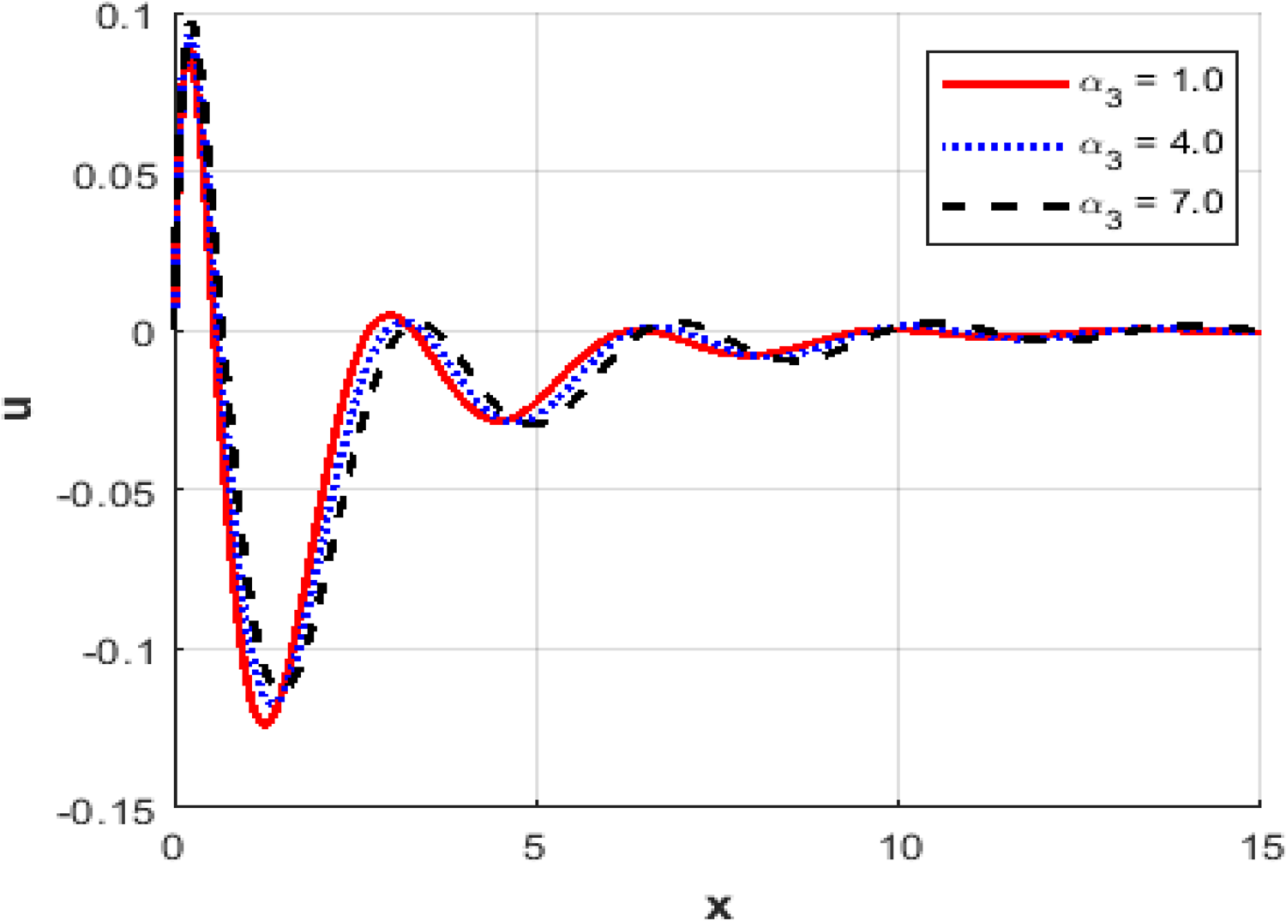
Variation of the displacement $u$ against $x$ for different values of thermo-elastic parameter.

**Fig. 10:**
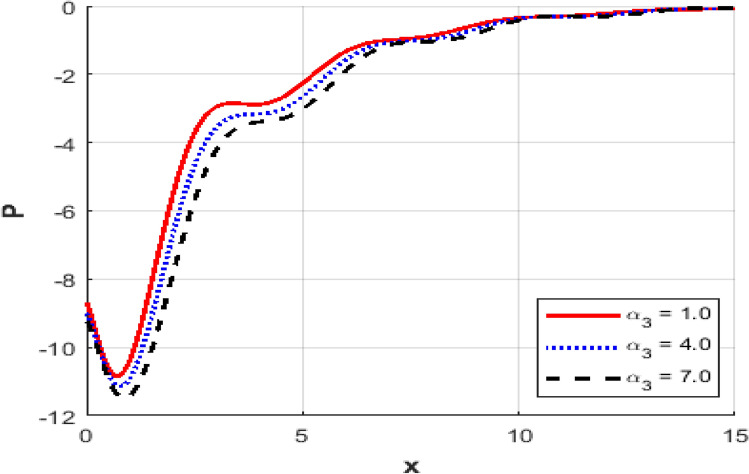
Variation of the acoustic pressure $P$ against $x$ for different values of thermo-elastic parameter.

**Fig. 11:**
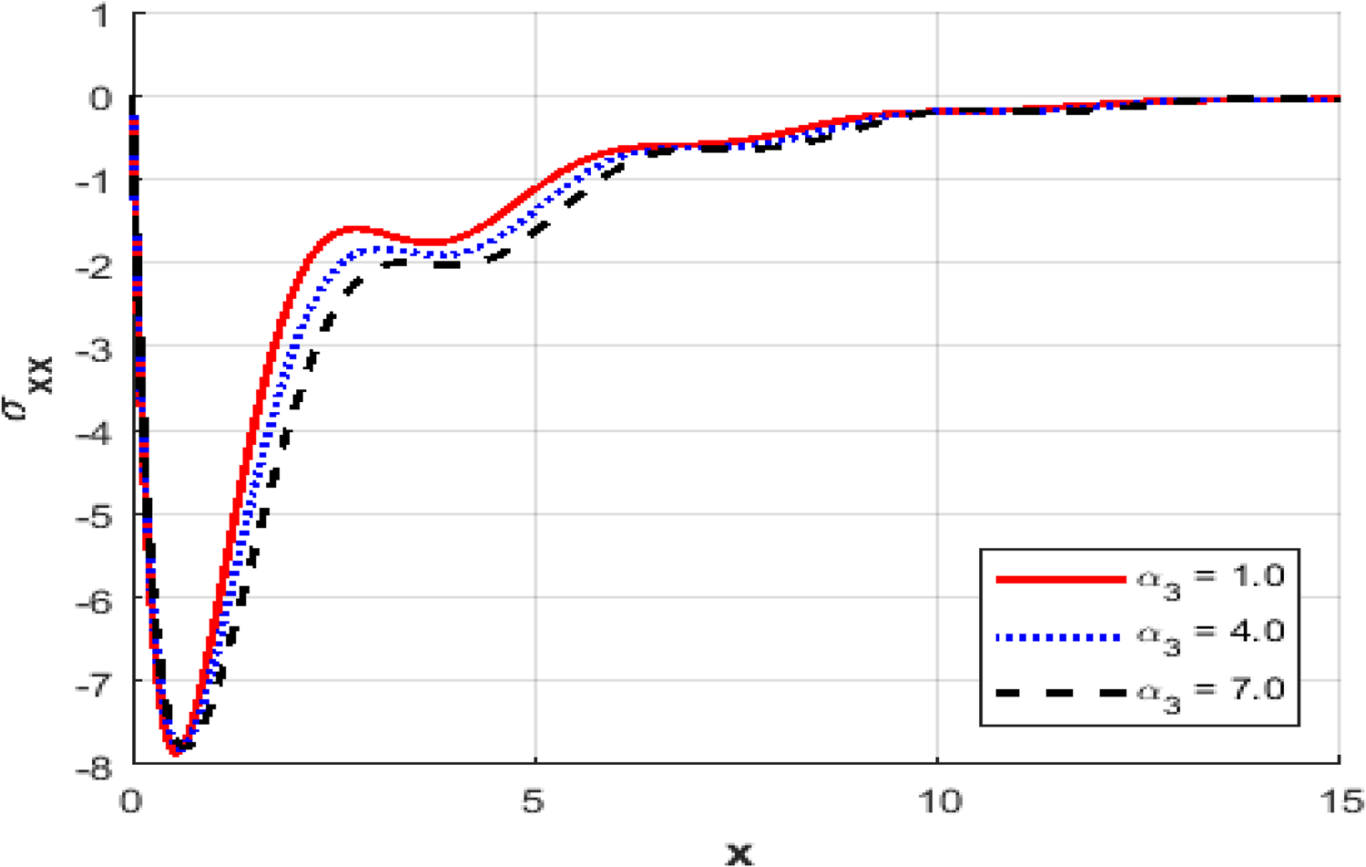
The stress distribution $\sigma_{xx}$ against $x$ for different values of thermo-elastic parameter.

**Fig. 12:**
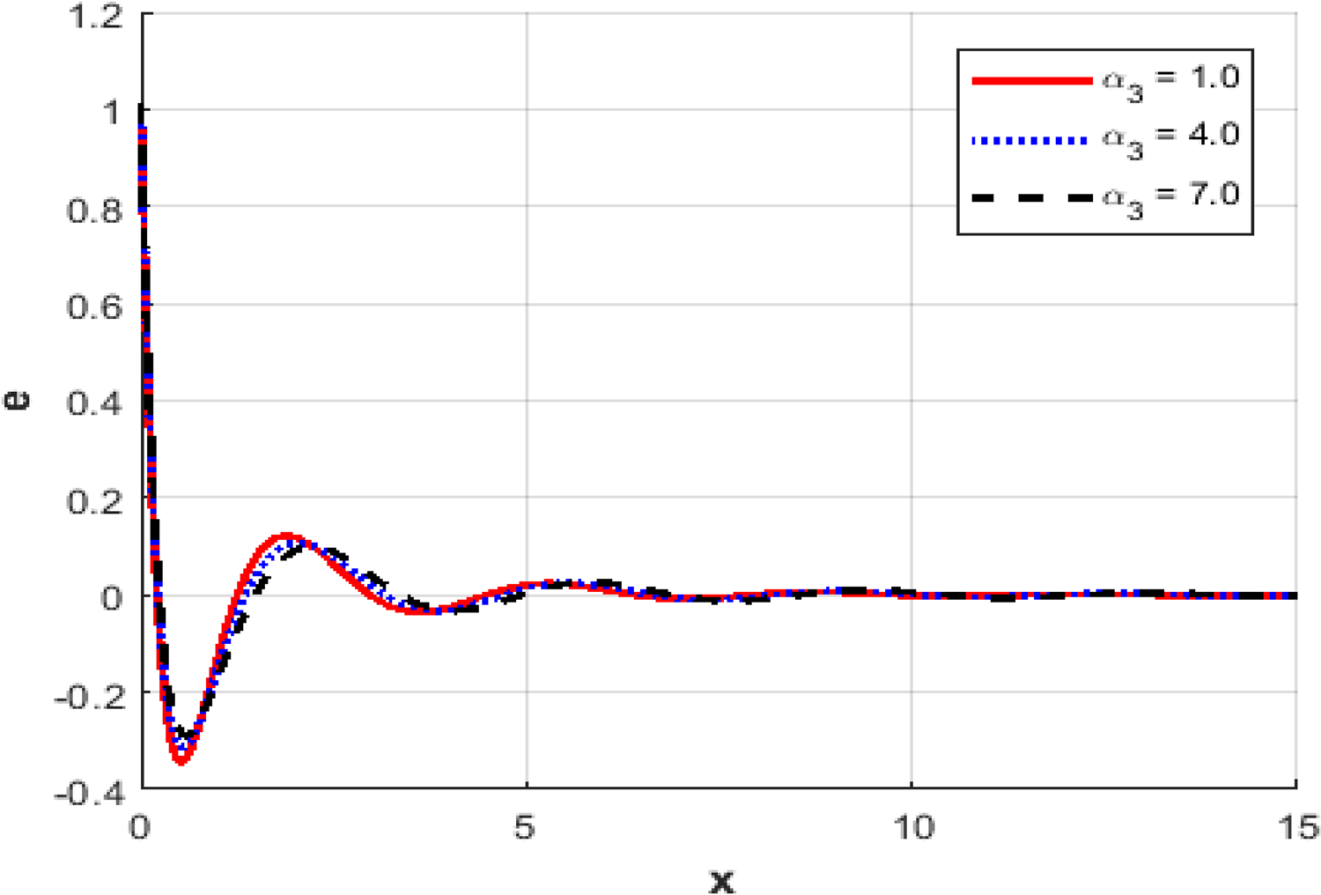
Variation of the strain $e$ against $x$ for different values of thermo-elastic parameter.

This section investigates the influence of varying the thermoelastic coupling parameter $\alpha_{3}$ on several key physical fields’ temperature, carrier density, displacement, acoustic pressure, stress, and strain within the framework of Moore-Gibson-Thompson theory during photo-acoustic semiconducting excitation. Figure 7 depicts the spatial distribution of temperature $T$ for various values of the thermoelastic coupling parameter $\alpha_{3} .$ Increasing the value of $\alpha_{3}$​ from $(0.1\,{\text{to}}\,0.7)$ results in a notable rise in the temperature peaks. The overall thermal wave amplitude is elevated, increasing the maximum temperature as $\alpha_{3}$​ grows. This behavior indicates enhanced thermoelastic interaction and energy transfer in response to stronger coupling. Figure 8 demonstrates the carrier density $N$ distribution, which exhibits an exponential decay irrespective of the thermoelastic coupling parameter. The curves corresponding to different $\alpha_{3}$​ values are indistinguishable, implying negligible dependence of the carrier density field on changes in thermoelastic coupling. This suggests that the coupling parameter variations under the given conditions minimally affect the electronic characteristics. The displacement field $u$ shown in Fig. 9 reveals oscillatory behavior close to the excitation boundary, followed by attenuation at more considerable distances. Increasing $\alpha_{3}$​ slightly modifies the amplitude of these oscillations, demonstrating minimal but discernible sensitivity of mechanical displacement to thermoelastic coupling strength. Oscillatory peaks become marginally smaller as $\alpha_{3}$​ increases, indicating slightly higher displacement damping. Figure 10 illustrates the acoustic pressure $P$ distribution, highlighting how increasing $\alpha_{3}$​ significantly affects the pressure profile. With larger coupling parameter values, deeper negative pressure minima are observed near the boundary. This suggests a direct relationship between thermoelastic coupling strength and the generated acoustic wave magnitude. The acoustic pressure gradually stabilizes to zero, far from the excitation boundary. The stress field $\sigma_{xx}$, presented in Fig. 11, mirrors the trends observed in the acoustic pressure profiles. Higher coupling parameter values $\alpha_{3}$​ lead to more pronounced negative stress peaks at the initial excitation region. These peaks indicate intensified stress due to enhanced thermoelastic interactions, after which stress diminishes and stabilizes at increased distances. Figure 12 provides the spatial distribution of strain $e$. Increasing $\alpha_{3}$ yields minor variations in strain, with the most significant differences occurring near the boundary where oscillations are evident. The strain quickly attenuates with distance, and the coupling parameter shows a limited influence on the magnitude of the strain oscillations, indicating moderate thermoelastic sensitivity compared to temperature and stress fields.

## Conclusion

This paper has extensively examined semiconductor materials subjected to thermo-acoustic and optical deformations within the sophisticated framework of the Moore-Gibson-Thompson Photo-Thermoelastic (MGPT) model. Our comprehensive investigation reveals that the thermoelectric and thermoelastic coupling parameters significantly influence various field variables, notably affecting temperature, displacement, and acoustic pressure. These parameters, however, exert only a slight effect on carrier density, suggesting nuanced sensitivity across different physical quantities. The theoretical and numerical analyses demonstrate pronounced peaks and rapid declines in temperature and acoustic pressure as the values of the thermo-electric coupling parameter increase. This indicates a sensitive and critical dependence on the coupling effect, particularly within the operational contexts of these materials. In contrast, the thermo-elastic coupling parameter showcases a stabilizing impact on displacement and temperature, reducing peaks as its value increases while also leading to significant declines in carrier density. This highlights its pivotal role in influencing semiconductors’ mechanical and thermal environments.

Moreover, the relationship between mechanical stress, strain, and the coupling parameter further underscores the intricate interplay within the material’s structural integrity. Lower parameter values correspond to higher stress and strain, defining semiconductor devices’ operational limits and durability under various loads.

All physical quantities rigorously satisfy the prescribed boundary conditions, attesting to the MGPT model’s robustness and reliability. The insights gained from this study enhance our theoretical understanding and pave the way for practical applications. These include improvements in semiconductor fabrication techniques and developing devices with improved performance in renewable energy and electronic manufacturing fields.

Ultimately, this research’s findings are integral to advancing the design and application of semiconductors in modern technologies, including solar cells, diodes, triodes, and other electronic devices. The potential for real-world application and relevance of these findings substantially contribute to the field, promising to future research and techno-logical innovations in semiconductor technology.

## Data Availability

The datasets used and/or analyzed during the current study available from the corresponding author on reasonable request.

## List of symbols

$$\lambda$$, $$\mu$$: Lame’s constants
$\delta_{n} = (3\lambda + 2\mu )d_{n}$: Deformation potential of conduction difference
$\gamma_{\theta } \, = (3\lambda + 2\mu )\alpha_{t}$: Volume thermal expansion
$d_{n}$: Electronic deformation potential
$\alpha_{t}$: Linear thermal expansion coefficient
$$\theta$$: Temperature absolute
$\theta_{0}$: The referencetemperature of the medium is assumed to be such that $\left| {(\theta - \theta_{0} )/\theta_{0} } \right| < 1$
$$N$$: Carrier intensity
${\varvec{r}}$: Vector of position
$u_{i}$: Vector of displacement
$\rho$: Density of mass
$C_{e}$: Specific heat at constant strain
$D_{E}$: The carrier coefficient of diffusion
$\tau$: The photo-generated carrier lifetime
$E_{g}$: The gap inenergy
$e_{ij}$: Strain tensor component
$\sigma_{ij}$: Stress tensor component
$$\delta_{ij}$$: Kronecker’s delta
$C_{s}$: The speed of sound in material
$C_{r}$: The adiabatic index or specific heat ratio of the material
$\beta$: Coefficient of volumetric thermal expansion of material
$\kappa = \frac{{\partial N_{0} }}{\partial \theta }\frac{\theta }{\tau }$: Parameter of thermal activation coupling
$N_{0}$: Concentration of carriers at equilibrium case
$$\upsilon$$: Thermal displacement
$$k^{ * }$$: Thermal conductivity
